# Histopathologic Improvement with Lymphedema Management, Léogâne, Haiti

**DOI:** 10.3201/eid1011.040548

**Published:** 2004-11

**Authors:** Susan F. Wilson, Jeannette Guarner, Alix L. Valme, Jacky Louis-Charles, Tara L. Jones, David G. Addiss

**Affiliations:** * Centers for Disease Control and Prevention, Atlanta, Georgia, USA;; † Hôpital Ste. Croix, Léogâne, Haiti

**Keywords:** Lymphatic filariasis, lymphedema, elephantiasis, histopathology, lymphedema management, limb care, women

## Abstract

In countries where bancroftian filariasis is endemic, lymphedema of the leg is a public health problem, particularly for women, who are disproportionately affected. We investigated the effect of basic lymphedema management (hygiene, skin care, and lower limb movement and elevation) on the histologic features of lymphedema. A total of 118 skin-punch biopsy specimens were collected from the legs of 91 patients enrolled in a lymphedema treatment clinic in Léogâne, Haiti. Follow-up biopsy specimens were collected from 27 patients ≈12 months later. Keratinocyte hyperproliferation, condensed dermal collagen, and mononuclear perivascular infiltrate increased with lymphedema stage, which suggested progressive chronic inflammation and fibrosis. Follow-up biopsies showed reductions in perivascular mononuclear infiltrate in the superficial dermis (41% decrease in prevalence), perivascular fibrosis in the deep dermis (58% decrease), and periadnexal mononuclear infiltrate (53% decrease). These data suggest that the clinical improvement commonly observed with basic lymphedema management has a histologic basis.

## Abstract 2

Basic management improves the histologic profile of limbs in patients with filarial lymphedema.

Lymphatic filariasis is an emerging disease in many areas of the tropics, where vector habitat has expanded because of large-scale water projects and declining sanitation associated with uncontrolled urban growth (*1**–**3*). In many countries where filariasis has been mapped systematically for the first time, its geographic distribution is much more extensive than previously believed (*4**,**5*). In Haiti, for example, the population at risk for infection was previously thought to be 1 million persons; however, the entire country (estimated population, 6–8 million) is now considered to be at risk (*5*).

Lymphedema of the limb is a physically deforming and socially stigmatizing consequence of filarial infection that affects ≈15 million persons worldwide (*6*). Although the factors responsible for the initiation and progression of filarial lymphedema to its most severe form, elephantiasis, have been debated, recurrent episodes of bacterial acute dermatolymphangioadenitis (ADLA) play a major role (*7**–**9*). Characterized by painful swelling of the limb, ADLA is accompanied by fever and chills lasting several days, sometimes with nausea and vomiting (*7**,**8**,**10*). As lymphedema progresses, the frequency of ADLA episodes generally increases (*11**,**12*). Skin changes of chronic lymphedema include thickening, nodular lesions, and pigmentary changes (*13**,**14*). Histopathologic studies have found evidence of inflammatory infiltrate in lymphedematous tissue (*14**,**15*).

Globally, lymphedema following infection with the filarial parasite *Wuchereria bancrofti* is more common in women than in men (*6**,**16**,**17*). In Haiti, the ratio of affected women to men is approximately 7 to 1 (*16*). Reasons for this discrepancy are unclear but may be related to differences in the "preferred" anatomic location of the adult filarial worm between men and women (*18*) and biologic factors, particularly pregnancy, that further stress the lymphatic system in women. Thus, in many filariasis-endemic areas, lymphedema is primarily a disease of women. Both the functional limitations caused by chronic lymphedema and the short-term impairment that accompanies episodes of ADLA compromise the ability of women to perform household chores and to participate in income-generating activities outside the home, which results in domestic and economic difficulties for their families and communities (*19**–**24*).

In 1998, the Global Program to Eliminate Lymphatic Filariasis embraced lymphedema management as a fundamental component of its strategy to eliminate lymphatic filariasis (*25*). Based on evidence of the bacterial etiology of ADLA, current World Health Organization (WHO) recommendations for management of lymphedema emphasize basic skin care and hygiene using soap, water, and antiseptics, as well as elevation of the leg, exercise, and proper footwear (*26*). Use of these measures improves skin condition, decreases the frequency of ADLA attacks, and reverses or arrests the progression of lymphedema, all of which improve quality of life (*24**,**27**–**29*).

Few studies in filariasis-endemic areas have examined stage-specific histologic changes in lymphedematous skin, and to our knowledge, no previous studies have examined histologic changes associated with WHO-recommended management of lymphedema. Our study attempts to characterize the histopathology of skin at different stages of lymphedema and assess histologic changes in the lymphedematous legs of patients enrolled in a lymphedema management program at Hôpital Ste. Croix in Léogâne, Haiti, an area where bancroftian filariasis is highly endemic.

## Materials and Methods

### Study Participants

The study protocol and consent forms were approved by the ethics committee at Hôpital Ste. Croix and the institutional review board at Centers for Disease Control and Prevention (CDC). Patients were eligible to participate if they were enrolled in the lymphedema treatment clinic at the hospital, had been examined by the clinic physician to rule out other causes of lower limb swelling, gave informed consent to collection of all biopsy specimens, had no medical contraindications to biopsy, had no ADLA episodes during the previous 2 weeks, and lived within a 10-km radius of the hospital.

Patients were tested for filarial infection by using an immunochromatographic card test (ICT), which detects antigen of adult *W. bancrofti* in the blood (*30*). Lymphedema stage was assessed by using an adaptation of a three-stage system recommended by WHO (*31*). Stage 1 lymphedema is characterized by swelling that is reversible on elevation at night. Stage 2 lymphedema is not reversible upon elevation and has no papillomatous changes. Stage 3 lymphedema, sometimes called elephantiasis, is characterized by papillomatous lesions and pronounced dermatosclerosis.

Patients were instructed in lymphedema self-care (*27*), with emphasis on thorough daily washing of the limb, basic skin care to treat and prevent entry lesions, range-of-motion limb exercises, and elevation of the leg during the day when possible and at night while sleeping. Participants were provided with basic supplies (e.g., soap, towels, wash basin) as needed. To monitor lymphedema self-care, patients returned to the clinic or were visited at home every 4–6 weeks and were asked about compliance with the regimen since the previous visit. Patients were encouraged to seek antimicrobial drugs and symptomatic treatment at the hospital during ADLA attacks. Thus, most ADLA attacks were observed by clinic staff; a few attacks were recorded on the basis of patient history and the presence of residual clinical signs (e.g., peeling of the skin, swelling) at the next follow-up visit.

### Biopsy and Analysis

A total of 91 patients agreed to undergo skin-punch biopsy of their lymphedematous leg or legs, and 26 of these patients also agreed to a biopsy of their non-lymphedematous leg (control). Twenty-seven patients agreed to have follow-up biopsies of their lymphedematous limb ≈12 months later. Biopsy specimens were taken from the anterolateral surface of the leg from a site that was representative in appearance of the rest of the leg. Irregular protrusions and skin lesions were avoided. After the skin was cleaned with betadine and anesthetized with 1% lidocaine, a 4-mm skin-punch biopsy specimen was obtained, and suture or 3M Steri-Strips were used to close the skin at the biopsy site. A short course of oral amoxicillin (250 mg three times a day for 5 days) was given to help prevent bacterial infection.

Skin-punch biopsy specimens were fixed in formalin in Léogâne, Haiti, and sent to CDC in Atlanta, Georgia, where they were embedded in paraffin. Five-micrometer sections were cut and stained with hematoxylin and eosin. All biopsy sections were read by the same pathologist (JG), who was blinded with respect to patient identification, lymphedema stage, and whether the specimen was from an initial or follow-up biopsy. Each biopsy specimen was evaluated for the presence or absence of the histopathologic characteristics presented in Table 1.

**Table 1:** Histopathologic features evaluated in skin biopsy specimens from patients with lymphedema of the leg, Léogâne, Haiti

Location, feature	Comments
Epidermis	
Hyperkeratosis	Thickening of horny layer, with disappearance of basket-weave pattern.
Hypergranulosis	Basophilic pyknotic nuclei in keratin layer.
Acanthosis	Increase in thickness of the stratum malpighii.
Superficial dermis	
Fibrolamellar hyperplasia	Distinct collagen bundles parallel to basal epidermal layer.
Condensed collagen	Thickened and closely packed collagen bundles with deep eosinophilic staining pattern.
Perivascular fibrosis	Condensed collagen concentric to vessels.
Perivascular infiltrate	Cellular infiltrate surrounding vessels, defined as either acute (presence of neutrophils or eosinophils) or chronic (presence of mononuclear inflammatory cells, including lymphocytes and macrophages). Intensity of chronic infiltrate was noted as mild (average of <5 lymphocytes or macrophages observed in 40x magnification viewing field of perivascular spaces) or pronounced (average of >5 lymphocytes or macrophages), based on examination of several fields per slide. Plasma cells noted.
Deep dermis and subcutaneous tissue	
Perivascular fibrosis	Same as in superficial dermis.
Perivascular infiltrate	Same as in superficial dermis.
Periadnexal infiltrate	Cellular infiltrate around hair, sweat, and sebaceous glands. Type and intensity were noted as previously defined for the superficial dermis.
Infiltrate in subcutaneous tissues	Cellular infiltrate in fibrous septa among adipose tissue. Type and intensity were noted as previously defined for superficial dermis.

### Statistical Analysis

Statistical analysis was performed using EpiInfo 6.0. The chi-square and Fischer exact tests were used to compare differences in the proportions of specimens with histopathologic features.

## Results

### Participants

Of the 91 patients enrolled in this study, 73 (80%) were female. Women and men did not differ significantly with regard to age, lymphedema stage, or histologic features (data not shown). Median age was 39 years (range 16–75 years). One patient had bilateral lymphedema; the others had unilateral disease. Two patients had filarial antigen detected in the blood by ICT. Both were treated with diethylcarbamazine, the drug of choice for *W. bancrofti* infection. The median length of time between enrollment in the lymphedema management program and the first biopsy was 21 days (range 0–866 days).

### Microbial Findings

No clinical signs of infection or inflammation were evident at the biopsy site. Microscopic examination of tissue sections stained with hematoxylin and eosin revealed no evidence of bacterial infection and no *W. bancrofti* adult worms or microfilariae.

#### Initial Biopsy Specimens and Histopathologic Features

One hundred eighteen biopsy specimens were collected, 92 from lymphedematous legs and 26 from nonlymphedematous control legs. Biopsy specimens were collected a median of 14 cm (range 5–20 cm) above the sole of the foot. The number of biopsy specimens taken from control legs and from legs with stage, 1, 2, or 3 lymphedema was 26, 12, 60, and 20, respectively. No postoperative infections developed. Among the biopsy specimens, the proportion with histopathologic features (prevalence) increased with lymphedema stage (Table 2).

**Table 2:** Number and percentage of initial skin biopsy specimens in which histopathologic features were detected, by stage of lymphedema, Léogâne, Haiti

Location in skin	Histopathologic characteristic	Control (N = 26) n (%)	Lymphedema		
			Stage 1 (N = 12) n (%)	Stage 2 (N = 60) n (%)	Stage 3 (N = 20) n (%)
Epidermis	Hyperkeratosis	4 (15)	5 (42)	23 (38)^a^	16 (80)^b^
	Hypergranulosis	0 (0)	1 (8)	5 (8)	4 (20)^a^
	Acanthosis	0 (0)	3 (25)^a^	11 (18)^a^	6 (30)^c^
Superficial dermis	Fibrolamellar hyperplasia	5 (19)	6 (50)	25 (42)^a^	11 (55)^a^
	Condensed collagen	0 (0)	1 (8)	18 (30)^c^	7 (35)^c^
	Perivascular fibrosis	0 (0)	1 (8)	6 (10)	3 (15)
	Perivascular infiltrate				
	Acute	0 (0)	0 (0)	2 (3)	0 (0)
	Chronic	11 (42)	8 (67)	46 (77)^c^	19 (95)^b^
	Pronounced intensity	1 (4)	2 (17)	11 (18)	7 (35)^c^
	Presence of plasma cells	0 (0)	0 (0)	9 (15)^a^	6 (30)^c^
Deep dermis	Perivascular fibrosis	3 (11)	1 (8)	16 (27)	8 (40)^a^
	Perivascular infiltrate				
	Acute	0 (0)	0 (0)	0 (0)	0 (0)
	Chronic	12 (46)	6 (50)	54 (90)^b^	19 (95)^b^
	Pronounced intensity	1 (4)	3 (25)	20 (33)^c^	11 (55)^b^
	Presence of plasma cells	1 (4)	7 (58)^b^	37 (62)^b^	15 (75)^b^
	Periadnexal infiltrate				
	Acute	0 (0)	0 (0)	0 (0)	0 (0)
	Chronic	4 (15)	4 (33)	36 (60)^b^	11 (55)^c^
	Pronounced intensity	0 (0)	0 (0)	10 (17)^a^	3 (15)
Subcutaneous tissue	Infiltrate in fibrous septa				
	Acute	0 (0)	0 (0)	0 (0)	0 (0)
	Chronic	1 (4)	3 (25)	19 (32)^c^	9 (45)^c^
	Pronounced intensity	1 (4)	3 (25)	5 (8)	3 (15)

^a^p < 0.05 (compared to control leg biopsy specimens). ^b^p < 0.001 (compared to control leg biopsy specimens). ^c^p < 0.01 (compared to control leg biopsy specimens).

#### Epidermis

The prevalence of hyperkeratosis, hypergranulosis, and acanthosis increased significantly with stage of lymphedema. Hyperkeratosis was the predominant pathologic feature within the epidermis, regardless of stage, and was found in 80% of biopsy specimens from legs with stage 3 lymphedema. No biopsy specimens from unaffected control legs showed hypergranulosis or acanthosis.

#### Superficial Dermis

Within the superficial dermis, the prevalence of fibrolamellar hyperplasia increased from 19% in nonlymphedematous legs to 50% in stage 1 lymphedema but remained similar for stages 1–3. The prevalence of condensed collagen steadily increased from 0% in unaffected control legs to 30% in stage 2 lymphedema (p = 0.002). Most of the perivascular infiltrate in the superficial dermis was composed of mononuclear cells, primarily lymphocytes and macrophages. Plasma cells were found only in stage 2 and 3 lymphedema and were significantly less common in biopsies from women (4 [4%] of 93) than from men (7 [28%] of 25) (p = 0.002). The prevalence of chronic mononuclear infiltrate increased steadily from 42% in nonlymphedematous biopsy specimens to 95% in stage 3 biopsy specimens (p = 0.0002). In all areas where cellular infiltrate was observed, lymphocyte and macrophage cell populations were assessed together with respect to infiltrate intensity because activated lymphocytes were difficult to differentiate microscopically from macrophages. The prevalence of pronounced lymphocyte and macrophage infiltrate increased with lymphedema severity: 4% of biopsies from nonlymphedematous legs had pronounced infiltrate compared to 35% of stage 3 biopsy specimens (p = 0.008).

#### Deep Dermis

Perivascular fibrosis was recorded in 27% and 40% of skin biopsy specimens from patients with stage 2 and stage 3 lymphedema, respectively; it was more prevalent within the deep dermis than the superficial dermis at nearly every stage of lymphedema. The prevalence of perivascular infiltrate, composed entirely of mononuclear cells, increased from 50% in stage 1 lymphedema to 90% in stage 2 (p = 0.003). Pronounced chronic infiltrate and plasma cells were also more common in the deep dermis than in the superficial dermis at every stage of lymphedema. The prevalence and intensity of periadnexal infiltrate, all of which was mononuclear, increased with stage of lymphedema. Plasma cells were rarely observed in periadnexal infiltrate.

#### Subcutaneous Tissue

All cellular infiltrate in fibrous septa surrounding subcutaneous adipose tissue was mononuclear, and its prevalence increased with lymphedema stage, particularly between nonlymphedematous controls and stage 1 lymphedema (4% to 25%). Plasma cells were rarely observed in the infiltrate surrounding subcutaneous tissue.

### Follow-up Participants and Biopsy Specimens

Follow-up biopsy specimens were collected from the lymphedematous limb of 27 patients a median of 365 days (range 317–656 days) after their first biopsy. Of these 27 patients, 20 (74%) were women; the median age was 38 years (range 16–61 years). They did not differ significantly by sex, age, or lymphedema stage from the 64 participants who only had one skin biopsy.

Compliance with self-care practices during the interval between biopsies was high. At 96%, 94%, 87%, and 98% of monthly follow-up visits, respectively, patients reported that, since the previous visit, they had washed the leg daily, practiced range-of-motion exercises, elevated the leg during the daytime, and raised the foot of the bed at night. No changes in lymphedema stage were observed.

The second biopsy specimen was obtained a median of 14 cm (range 6–17 cm) above the sole of the foot, and a median of 1 cm (range <1–5 cm) from the first biopsy site. No postoperative infections developed. Of the 27 biopsy specimens that were collected for follow-up, 2 were from legs with stage 1 lymphedema, 18 from stage 2 lymphedema, and 7 from stage 3 lymphedema.

Of the 27 patients, 21 (78%) had reported one or more ADLA attacks during the 12-month period before entering the program. In contrast, only eight (30%) reported one or more attacks during the interval between biopsies (1, 4, and 3 patients with stage 1, 2, and 3 lymphedema, respectively). The mean reported incidence of attacks during the year before entering the program was 1.7 (range, 0–8) per person-year, compared to 0.5 (range, 0–3) observed between biopsies (p = 0.0009).

### Histopathologic Changes with Lymphedema Management

**Figure 1:**
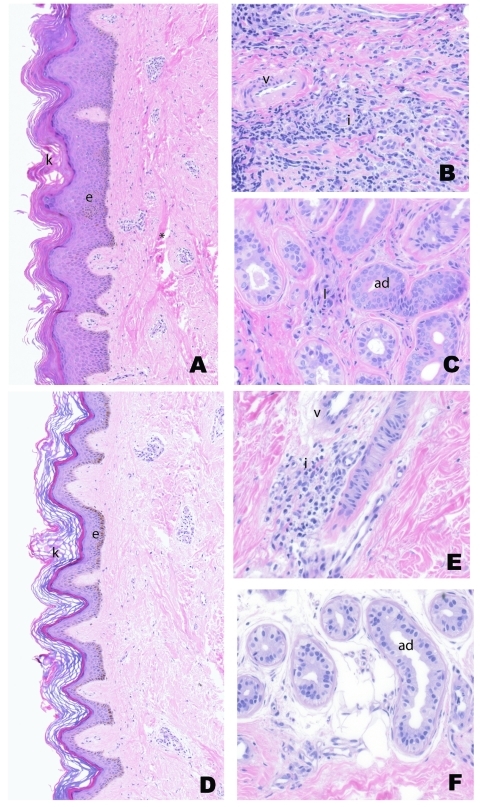
Representative sample of skin punch biopsy specimens from patients with lymphedema before (A, B, C) and after (D, E, F) 1 year of basic lymphedema management. Pretreatment abnormalities of the epidermis (e), which include increased number of epithelial cells (acanthosis and epidermal hyperplasia) and thickening of the keratin (k) layer, were improved after treatment (compare first [A] and second [D] biopsy specimens from same patient). Also noted is thickening of collagen bundles (*) in the dermis on the first (A) sample, which is not observed on the second (D). The intensity of inflammatory cells (i), which stain blue, surrounding fibrosed vessels (v) on the first sample (B), are more prominent than in the second sample (E). The amount of inflammation (i) in the subcutaneous adipose tissue is also more pronounced in the first biopsy sample (C) than the second sample (F) where adnexa (ad) with minimal inflammation can be observed. (Hematoxylin and eosin stains; original magnification for A, C, D, and F = 25x; B and E = 50x.)

The prevalence and intensity of histopathologic abnormalities tended to be greater in initial skin specimens (Figure 1A, B, and C) than in follow-up specimens (Figure 1D, E, and F) (Table 3). Hyperkeratosis and hyperplasia of the epidermis were more prominent on initial specimens (Figure 1A) than on follow-up specimens (Figure 1D). The thick collagen bundles observed in the dermis of initial biopsy samples (Figure 1A) were less obvious in specimens after 1 year of lymphedema management (Figure 1D). In the superficial dermis, substantial decreases were observed between the first (Figure 1B) and second (Figure 1E) biopsies, both in the prevalence of chronic perivascular infiltrate (100% to 59%, p = 0.0002, Table 3) and in the proportion of specimens with pronounced infiltrate intensity (37% to 11%, p = 0.03).

**Table 3:** Number and percentage of skin biopsy specimens in which histopathologic features were detected in 27 patients practicing lymphedema management, Léogâne, Haiti^a^

Location in skin	Histopathologic characteristic	1st biopsy (N = 27) n (%)	2nd biopsy (N = 27) n (%)	p value
Epidermis	Hyperkeratosis	14 (52)	12 (44)	0.59
	Hypergranulosis	5 (18)	1 (4)	0.09
	Acanthosis	7 (26)	3 (11)	0.16
Superficial dermis	Fibrolamellar hyperplasia	16 (59)	11 (41)	0.18
	Condensed collagen	10 (37)	5 (18)	0.13
	Perivascular fibrosis	4 (15)	1 (4)	0.17
	Perivascular infiltrate			
	Acute	0 (0)	0 (0)	
	Chronic	27 (100)	16 (59)	0.0002
	Pronounced intensity	10 (37)	3 (11)	0.03
	Presence of plasma cells	7 (26)	4 (15)	0.31
Deep dermis	Perivascular fibrosis	14 (52)	6 (22)	0.02
	Perivascular infiltrate			
	Acute	0 (0)	0 (0)	
	Chronic	25 (93)	21 (78)	0.12
	Pronounced intensity	9 (33)	6 (22)	0.37
	Presence of plasma cells	18 (67)	18 (67)	1
	Periadnexal infiltrate			
	Acute	0 (0)	0 (0)	
	Chronic	19 (70)	9 (33)	0.007
	Pronounced intensity	7 (26)	0 (0)	0.005
Subcutaneous tissue	Infiltrate in fibrous septa			
	Acute	0 (0)	0 (0)	
	Chronic	10 (37)	10 (37)	1
	Pronounced intensity	5 (18)	1 (4)	0.09

^a^For each patient, the second biopsy specimen was taken near the same site on the same lymphedematous leg ≈1 year after the first biopsy.

Perivascular fibrosis in the deep dermis was less common in follow-up biopsy samples (22%, Figure 1E) than in initial samples (52%, Figure 1B). Additionally, significant decreases were observed in the prevalence of chronic periadnexal infiltrate (70% to 33%, p = 0.007) and the percentage of specimens with pronounced infiltrates of lymphocytes and macrophages in periadnexal areas of the deep dermis (26% to 0%, p = 0.005, Table 3, and Figure 1C and F). The replacement of thick collagen bundles around adnexi in the first specimen (Figure 1C) with adipose cells in the second specimen (Figure 1F) was also noted in the subcutaneous tissue.

**Figure 2:**
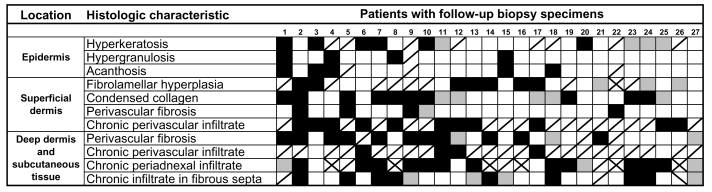
Individual histologic responses of the 27 patients with follow-up biopsy specimens who participated in a lymphedema management program for ≈1 year. Black boxes indicate histopathologic regression or improvement, gray boxes indicate histopathologic progression or worsening, white boxes indicate the absence of histopathologic changes in either biopsy specimens, and boxes with a diagonal line indicate that histopathologic changes were observed on both initial and follow-up biopsy specimens. Boxes with an X indicate insufficient data.

Figure 2 summarizes the histologic changes in each of the 27 patients with follow-up biopsies. Histopathologic regression or improvement was defined as the disappearance of a histopathologic characteristic on follow-up specimen, while histopathologic progression or worsening was defined as the appearance of a previously unnoted histopathologic characteristic on follow-up specimen. All but one patient (96%) showed histopathologic regression in one or more characteristics. Histopathologic improvement was observed among all stages of lymphedema. However, 4 (57%) of 7 legs with stage 3 lymphedema showed regression of chronic perivascular infiltrate in the deep dermis, compared to none of 20 legs with stage 1 or 2 lymphedema (p = 0.003). Eleven (41%) patients showed regression in four or more characteristics; they did not differ significantly from the other 16 patients with respect to sex, age, stage, or duration of lymphedema; duration of participation in the lymphedema program before the first biopsy; interval between biopsies; or ADLA incidence between biopsies (Table 4). Sixteen (59%) patients showed histopathologic progression in one or more characteristics. None of the 27 patients had progression of chronic perivascular infiltrate.

**Table 4:** Demographic, physical, and treatment-related characteristics of 27 patients with initial and follow-up skin biopsy specimens, by degree of histologic improvement (involving >4 vs. <4 histologic characteristics), Léogâne, Haiti.

Factors examined	Improvement in >4 histologic characteristics	Improvement in <4 histologic characteristics
Number of patients	11	16
Female (%)	9 (82)	11 (69)
Median age, y (range)	39 (20–61)	32 (16–61)
Stage of lymphedema (%)		
Stage 1	2 (18)	0 (0)
Stage 2	5 (45)	13 (81)
Stage 3	4 (36)	3 (19)
Median no. of days in lymphedema management before 1st biopsy (range)	21 (0–432)	19.5 (0–468)
Median no. of days between biopsies (range)	359 (317–503)	366 (317–656)
Median annual incidence of ADLA between biopsies (range)	0 (0–2)	0 (0–3)
Median duration of lymphedema, y (range)	11.5 (4–27)	7 (0–25)

## Discussion

The physical, personal, social, and economic difficulties caused by lymphedema and elephantiasis of the leg in many filariasis-endemic areas disproportionately affect women (*6**,**16**,**17**,**21**,**24*). Filariasis elimination programs' increasing adoption of simple, inexpensive measures for lymphedema management has led to reduced ADLA incidence, reduced lymphedema-related illness, decreased stigma, and improved quality of life for women in many filariasis-endemic areas (*24**,**27**–**29**,**32*). Our study complements these findings by providing evidence of histologic improvement in patients who routinely practice lymphedema self-care.

Increasing lymphedema stage was associated with increased proliferation of keratinocytes and acanthosis in the epidermis and with an increased prevalence of fibrolamellar hyperplasia, condensed collagen, perivascular fibrosis, and perivascular and periadnexal infiltrate in the dermis. Epidermal hyperproliferation, which has been described previously for lymphedema in filariasis-endemic areas (*14*), can result from an influx of macrophages that release epidermal growth factors in response to repeated irritation caused by foreign antigens within the skin.

The condensed collagen bundles and perivascular fibrosis in our biopsy specimens help explain the hardness of the skin in persons with stage 3 lymphedema. We observed a progression from thin, lacelike dermal collagen in biopsy samples from legs with no lymphedema to fibrolamellar hyperplasia in the rete ridges accompanied by thick condensed collagen bundles in patients with severe lymphedema. In some cases, these collagen bundles encased vascular vessels as well as adnexal structures. Lymph stasis, which results from lymphatic dysfunction, leads to accumulation of blood proteins, cellular metabolic products, and recirculating lymphocytes in the tissue (*33*). The presence of these molecules and cells has been shown to induce epidermal thickening, deposition of collagen in the dermis, and proliferation of fibroblasts (*33**,**34*).

The prevalence of mononuclear inflammatory cells around vessels and adnexa and in the fibrous septa also increased with lymphedema stage. The inflammatory infiltrate consisted predominantly of lymphocytes and macrophages, similar to what has been previously reported (*14**,**15*). The high frequency of plasma cells in the deep dermis, present in nearly 60% of stage 1 specimens, was unusual and has not been previously reported in patients with lymphedema in filariasis-endemic areas. Plasma cells are infrequently found in healthy skin and are generally associated with chronic inflammation or bacterial infections (*35*). The dramatically higher number of plasma cells within the deep dermis suggests a more pronounced immunologic response than in the superficial dermis.

The stimulus for chronic mononuclear infiltrate within the superficial and deep dermis in these patients is unknown. Chronic infiltrate may have resulted from poor clearance by the lymphatic system of bacteria penetrating the skin surface or a prolonged immune response following an ADLA attack. Chronic inflammation might also be provoked by the presence of macromolecules, the production of cytokines and growth factors, and their accumulation in the skin. Studies in filariasis-endemic areas have shown proinflammatory immune proteins and cytokines in the serum and lymph fluid of patients with lymphedema (*34*).

The fact that specimens from some nonlymphedematous legs had histologic abnormalities, especially chronic perivascular infiltrate, has several possible explanations. First, these abnormalities may have been due to minor leg trauma (i.e., cuts and bruises) or interdigital fungal infections (*27*). Of 11 biopsy specimens from nonlymphedematous legs that had perivascular lymphocytes and macrophages in the superficial dermis, only 1 (9%) showed pronounced infiltrate. Second, stage 1 lymphedema may have been misclassified as nonlymphedematous in some cases. Finally, subclinical damage may have already been present in the legs that appeared normal.

A trend toward improvement was noted for virtually all histologic characteristics examined in the follow-up biopsy specimens. The marked improvement in cellular infiltrate in the superficial dermis is consistent with the effect of improved skincare and hygiene. In addition, the prevalence and intensity of chronic cellular infiltrate surrounding adnexa were significantly reduced during the 1-year period. Taken as a whole, these observations are consistent with the hypothesis that basic lymphedema management, which reduced microbial load on the skin surface and healed entry lesions, led to a decrease in ADLA incidence and a reduction in chronic inflammation of the skin.

No changes were observed in lymphedema stage despite reductions in skin inflammation and fibrosis; however, the three-stage classification system for lymphedema provides only a gross assessment of clinical status. A seven-stage system with better discriminating power (*27*), developed after this study was completed, is currently being used in filariasis-endemic areas.

At an individual level, we found no factors, including the absence of ADLA attacks, that were significantly associated with histopathologic regression. This finding may be attributed to the limited number of persons in each group, the prompt use of antimicrobial drugs after onset of ADLA symptoms, or variation in inflammatory responses among persons. Acute histologic responses to a single ADLA attack also may have been transient, so that by the time of the second biopsy, histologic markers of the episode had cleared.

This study has several limitations. First, no control group was included, since ethical considerations precluded collecting follow-up biopsies from persons not instructed in lymphedema self-care. However, we would not have expected to observe significant histopathologic improvement in the absence of intervention. Second, we did not use special stains for bacteria or immunohistochemical assays for subtyping cells and collagen, all of which would be useful for understanding the pathogenesis of lymphedema and are currently planned. Third, the interval between initiating lymphedema self-care and the first biopsy varied among patients; the first biopsy specimen was not always a "baseline" specimen. However, this variation did not appear to influence the degree of histologic improvement during follow-up, which suggests that the benefits of lymphedema management are not limited to the first few months but continue to accrue with practice. Finally, the number of patients included in the follow-up study was small, which limited statistical power. Larger studies, preferably involving several centers, are recommended.

In conclusion, participation in a lymphedema management program for 1 year was associated with significant reductions in cellular infiltrate and fibrosis. Lymphedema management, based on inexpensive and practical elements of self-care at home, can lead not only to histologic improvement, as shown here, but also to clinical and functional benefits and to improved quality of life. Programs in Brazil (*27*), India (*29*), Haiti (*24**,**32*), Guyana (*28*), and elsewhere have documented these benefits and pioneered creative ways, such as support groups (*32*), to teach affected women the principles of lymphedema self-care and motivate them to continue to practice it. In most filariasis-endemic areas, however, such programs do not yet exist. To reach the millions of women who suffer from this disease, lymphedema management must be expanded, as an integral part of the Global Program to Eliminate Lymphatic Filariasis, to all major filariasis-endemic areas worldwide.
